# Multicenter Point-Prevalence Study of Carbapenem-Resistant *Klebsiella* spp. in Türkiye: Epidemiology, Antimicrobial Resistance, and Infection Control Practices

**DOI:** 10.3390/antibiotics15070679

**Published:** 2026-07-10

**Authors:** Esra Erdem Kıvrak, Deniz Özer, Sinan Mermer, Ahmet Sertçelik, Hasip Kahraman, Müge Toygar Deniz, Damla Ertürk, Arzu Nazlı, Hüseyin Aytaç Erdem, Ongun Yeniçeri, Meltem Taşbakan

**Affiliations:** 1 Department of Infectious Diseases and Clinical Microbiology, Faculty of Medicine, Manisa Celal Bayar University, Manisa 45140, Türkiye; dnztrk78@gmail.com; 2 Department of Infectious Diseases and Clinical Microbiology, Faculty of Medicine, İzmir University of Economics Medical Point Hospital, İzmir 35100, Türkiye; sinan.mermer@ieu.edu.tr; 3 Department of Infectious Diseases and Clinical Microbiology, Division of Epidemiology, Faculty of Medicine, Ankara Yıldırım Beyazıt University, Ankara Bilkent City Hospital, Ankara 06800, Türkiye; ahmetsertcelik@gmail.com; 4 Department of Infectious Diseases and Clinical Microbiology, Faculty of Medicine, Eskişehir Osmangazi University, Eskişehir 26480, Türkiye; hasipkahraman@gmail.com; 5 Department of Infectious Diseases and Clinical Microbiology, Faculty of Medicine, Kocaeli University, Kocaeli 41380, Türkiye; mugedeniz90@gmail.com; 6 Department of Infectious Diseases and Clinical Microbiology, Faculty of Medicine, Çukurova University, Adana 01330, Türkiye; damlaerdogan_@hotmail.com (D.E.); ongun448@hotmail.com (O.Y.); 7 Department of Infectious Diseases and Clinical Microbiology, Faculty of Medicine, Dokuz Eylül University, İzmir 35100, Türkiye; arzu.nazli@deu.edu.tr; 8 Department of Infectious Diseases and Clinical Microbiology, Faculty of Medicine, Ege University, İzmir 35100, Türkiye; huseyin.aytac.erdem@ege.edu.tr

**Keywords:** carbapenem resistance, *Klebsiella* spp., point prevalence, healthcare-associated infection, antimicrobial resistance, infection control

## Abstract

Background: Carbapenem-resistant *Klebsiella* spp. have emerged as major healthcare-associated pathogens worldwide, posing significant therapeutic and infection control challenges. Despite the growing burden of antimicrobial resistance, contemporary multicenter epidemiological data regarding carbapenem-resistant *Klebsiella* spp. in Türkiye remain limited. This study aimed to determine the culture-based prevalence of carbapenem-resistant *Klebsiella* spp. and to describe the clinical, microbiological, and institutional characteristics associated with these organisms. Materials and Methods: This multicenter cross-sectional point-prevalence study was conducted in seven tertiary-care hospitals located in different regions of Türkiye. On 19 February 2026, all hospitalized adult patients and microbiological cultures obtained on the study day were evaluated. Demographic, clinical, microbiological, antimicrobial treatment, infection control, and hospital-level data were collected using a standardized case report form. Culture-based prevalence estimates were calculated with 95% confidence intervals (CIs). Results: A total of 26 patients with carbapenem-resistant *Klebsiella* spp. colonization and/or infection were included. The mean age was 68.5 ± 14.1 years, and 61.5% were male. Most patients had at least one comorbidity (84.6%), and recent antibiotic exposure was common (88.5%). Urinary tract infection was the most frequently identified infection type (53.8%), followed by pneumonia (19.5%) and bloodstream infection (19.2%). Urinary catheterization was present in 73.1% of patients, while central venous catheters and mechanical ventilation were present in 46.2% and 34.6%, respectively. Ceftazidime–avibactam susceptibility results were available for 20 isolates, of which 12 (60.0%; 95% CI, 38.66–78.12) were resistant. Colistin susceptibility results were available for 17 isolates, of which four (23.5%; 95% CI, 9.56–47.26) were resistant. The culture-based prevalence of carbapenem-resistant *Klebsiella* spp. was 1.69% (95% CI, 1.16–2.46) among all cultures obtained on the study day and 8.94% (95% CI, 6.17–12.77) among culture-positive specimens. Conclusions: Carbapenem-resistant *Klebsiella* spp. remain important healthcare-associated pathogens in Türkiye, particularly among patients with substantial healthcare exposure, invasive device use, and recent antibiotic treatment. Continued surveillance, strengthened infection prevention measures, and expanded molecular characterization are essential for improving the understanding and control of antimicrobial resistance in the country.

## 1. Introduction

Antimicrobial resistance (AMR) is one of the greatest threats to global public health and has become a major challenge for modern healthcare systems. According to the 2024 World Health Organization (WHO) Bacterial Priority Pathogens List, carbapenem-resistant *Klebsiella pneumoniae* is classified as a critical priority pathogen because of its high attributable mortality, rapid dissemination, and limited therapeutic options [1]. Furthermore, recent surveillance data from the European Centre for Disease Prevention and Control (ECDC) demonstrated a continued increase in carbapenem-resistant *K. pneumoniae* bloodstream infections across Europe, highlighting the urgent need for continuous surveillance, effective infection prevention and control measures, and robust antimicrobial stewardship programs [2].

Enterobacterales are among the most frequently isolated Gram-negative pathogens in both community and healthcare settings. The development of resistance to broad-spectrum antibiotics such as carbapenems poses substantial challenges in the clinical management of critically ill patients. Infections caused by these microorganisms have been associated with increased mortality, prolonged hospitalization, and adverse clinical outcomes [3]. Furthermore, carbapenem-resistant Enterobacterales (CRE) isolates frequently exhibit multidrug-resistant phenotypes and may demonstrate resistance to multiple antimicrobial classes. Although the introduction of novel β-lactam/β-lactamase inhibitor combinations, including ceftazidime–avibactam, meropenem–vaborbactam, and imipenem–relebactam, has expanded available treatment options, the emergence of resistance to these agents remains a significant concern [4]. Among carbapenem-resistant Enterobacterales, *Klebsiella pneumoniae* represents the predominant pathogen in healthcare-associated infections and accounts for a substantial proportion of CRE-related morbidity and mortality worldwide [1]. Türkiye is considered one of the countries where OXA-48-like carbapenemases are endemic, making continuous surveillance of carbapenem-resistant *Klebsiella* spp. particularly important for guiding empirical antimicrobial therapy, infection prevention policies, and antimicrobial stewardship programs [2].

Intensive care units represent high-risk environments for the emergence and dissemination of antimicrobial-resistant microorganisms because of the frequent use of invasive procedures, prolonged hospital stays, and extensive exposure to broad-spectrum antibiotics. In this context, carbapenem-resistant Gram-negative bacteria (CR-GNB) are among the most important pathogens contributing to increased mortality in critically ill patients. Carbapenemase production constitutes one of the principal resistance mechanisms in these organisms, while horizontal transfer of resistance genes accelerates their dissemination. The global spread of carbapenemases, particularly KPC, NDM, OXA-48, and VIM, has further emphasized the importance of effective surveillance systems, infection prevention and control measures, and antimicrobial stewardship programs [5].

Carbapenem-resistant *Klebsiella pneumoniae* (CRKP) causes a broad spectrum of healthcare-associated infections, particularly in intensive care units, including pneumonia, bloodstream infections, sepsis, urinary tract infections, and surgical site infections. Owing to limited therapeutic options and substantial mortality rates, CRKP remains a major challenge for contemporary clinical practice and infection prevention efforts [6]. Therefore, understanding regional epidemiological patterns and associated clinical characteristics is essential for guiding infection prevention strategies and optimizing empirical antimicrobial therapy.

Although several studies from Türkiye have evaluated the antimicrobial susceptibility profiles or clinical outcomes of carbapenem-resistant *Klebsiella*, contemporary multicenter point-prevalence data integrating epidemiological, microbiological, and institutional characteristics across different geographical regions of the country remain limited. Therefore, the present multicenter point-prevalence study aimed to determine the culture-based prevalence of carbapenem-resistant *Klebsiella* spp. across tertiary-care hospitals in different regions of Türkiye and to characterize the epidemiological, clinical, microbiological, and institutional characteristics of colonized and/or infected patients.

## 2. Results

A total of 26 patients were included in the study. Sixteen patients (61.5%) were male, and the mean age was 68.5 ± 14.1 years. At least one comorbidity was present in 84.6% of patients. The most common comorbid conditions were coronary artery disease (42.3%), diabetes mellitus (38.5%), and chronic kidney disease (23.1%). Patients were hospitalized in surgical wards (42.3%), medical wards (38.5%), and intensive care units (19.5%). The median length of hospital stay was 21.5 days (interquartile range [IQR], 2.8–48.3). A history of hospitalization within the previous 90 days was reported in 42.3% of patients, while 30.8% had a history of intensive care unit admission during the same period. Recent antibiotic exposure within the preceding 30 days was documented in 88.5% of patients (Table 1).

Urinary tract infection (53.8%) was the most frequently identified infection type, followed by pneumonia (19.5%) and bloodstream infection (19.2%). Overall, 80.8% (21/26) of cases were classified as healthcare-associated infections according to the ECDC case definitions, whereas the remaining three cases did not meet these criteria (Table 2) [7].

Among microbiological specimens, urine samples were the most commonly collected (53.8%), followed by respiratory specimens (26.9%) and blood cultures (19.2%) (Table 3).

A total of 28 clinical specimens were obtained from 26 patients. Because the unit of analysis was the patient, percentages were calculated using the number of patients (*n* = 26). One patient contributed both blood and urine specimens, whereas another contributed both respiratory and wound specimens.

The use of invasive medical devices was common among the study population. Urinary catheters were present in 73.1% of patients, central venous catheters in 46.2%, and mechanical ventilation in 34.6%. The median duration of device use was 23.0 days (IQR, 8.0–30.0) for urinary catheters, 21.0 days (IQR, 9.3–55.0) for central venous catheters, and 25.0 days (IQR, 8.0–73.5) for mechanical ventilation. Among the 23 patients who received antimicrobial therapy, 60.9% received targeted treatment, whereas 39.1% received empirical treatment (Table 4).

Isolation precautions were implemented in 92.3% of patients, with contact precautions being the most frequently applied strategy (65.4%). Carbapenem-resistant *Klebsiella* spp. were considered the causative pathogens in 24 patients. All isolates were classified as multidrug-resistant (MDR). Ceftazidime–avibactam susceptibility results were available for 20 isolates, of which 12 (60.0%; 95% CI, 38.66–78.12) were resistant. Colistin susceptibility results were available for 17 isolates, of which four (23.5%; 95% CI, 9.56–47.26) were resistant. The molecular mechanism responsible for carbapenem resistance could be identified in only one isolate, in which KPC production was detected (Table 5).

All seven participating centers were tertiary-care university or university-affiliated teaching hospitals located in different geographical regions of Türkiye (Table 6). The number of carbapenem-resistant *Klebsiella* spp. cases identified per center ranged from two to six. Hospital characteristics, microbiological workload, and infection prevention practices varied across centers. Routine screening for CRE was implemented in two centers, whereas routine isolation precautions for patients with resistant microorganisms were reported by seven centers. An antimicrobial stewardship program was available in six of the seven participating hospitals. Although the culture-based prevalence of carbapenem-resistant *Klebsiella* spp. varied among centers, the limited number of cases precluded meaningful statistical comparisons.

The median adult bed capacity of participating centers was 731 beds (IQR, 678–986), whereas the median intensive care unit capacity was 83 beds (IQR, 53–126). The culture-based prevalence of carbapenem-resistant *Klebsiella* spp. among all cultures obtained on the study day was 1.69% (95% CI, 1.16–2.46). Among culture-positive specimens, the culture-based prevalence was 8.94% (95% CI, 6.17–12.77) (Table 6).

Hospital-level characteristics and infection prevention practices of the participating centers are summarized in Table 7. The number of carbapenem-resistant *Klebsiella* spp. cases identified per center ranged from two to six, and the culture-based prevalence among culture-positive specimens ranged from 5.3% to 25.0%. Although standard infection prevention measures were implemented across most participating centers, variability was observed in routine CRE screening policies and antimicrobial stewardship program implementation. Because of the limited number of cases identified at each participating center, formal statistical comparisons between hospitals were not performed.

## 3. Discussion

In this multicenter point-prevalence study, we evaluated the occurrence of carbapenem-resistant *Klebsiella* spp. and their associated clinical characteristics across tertiary-care hospitals located in different regions of Türkiye. The culture-based prevalence of carbapenem-resistant *Klebsiella* spp. was 1.69% among all cultures obtained on the study day and 8.94% among culture-positive specimens. Furthermore, the high proportion of healthcare-associated infections, frequent recent antibiotic exposure, and widespread use of invasive medical devices indicate that carbapenem-resistant *Klebsiella* spp. continue to represent a significant nosocomial pathogen in contemporary healthcare settings.

Although the number of identified cases was limited, the primary objective of this study was not to estimate incidence but rather to provide a multicenter snapshot of the epidemiology of carbapenem-resistant *Klebsiella* spp. across tertiary-care hospitals in Türkiye. The inclusion of geographically distinct centers enhances the epidemiological relevance of the findings and provides insight into the current national burden of these pathogens.

Previous systematic reviews and meta-analyses have demonstrated substantial regional variation in the prevalence of carbapenem-resistant *Klebsiella pneumoniae* infections, particularly among healthcare-associated infections [8]. The rapid global dissemination of carbapenemase-producing *K. pneumoniae* has further increased the clinical significance of these pathogens [9,10]. Although direct comparison with international studies is challenging because of methodological differences, our findings confirm that carbapenem-resistant *Klebsiella* spp. remain an important clinical and public health concern in Türkiye.

Although the single-day design may not fully capture temporal fluctuations in antimicrobial resistance epidemiology, it represents a pragmatic and standardized approach for multicenter surveillance and provides a valuable snapshot of the contemporary epidemiological situation.

The advanced age of the study population and the high prevalence of comorbid conditions are consistent with previous reports describing patients with carbapenem-resistant *K. pneumoniae* infections. Coronary artery disease, diabetes mellitus, and chronic kidney disease were the most frequently observed comorbidities. Increasing chronic disease burden is known to increase healthcare exposure and the risk of colonization or infection with multidrug-resistant organisms [11,12].

Recent healthcare exposure was common in the present cohort. Nearly half of the patients had been hospitalized within the previous 90 days, one-third had a history of intensive care unit admission, and almost 90% had received antibiotics during the preceding month. Previous hospitalization and antimicrobial exposure are well-established risk factors for carbapenem-resistant Enterobacterales colonization and infection [13]. The remarkably high rate of recent antibiotic use observed in our study further supports the role of antimicrobial selective pressure in the emergence and persistence of resistant pathogens.

Urinary tract infection was the most common clinical presentation, followed by pneumonia and bloodstream infection. Similar findings have been reported in previous studies, where carbapenem-resistant *K. pneumoniae* has been recognized as an important cause of complicated urinary tract infections and severe invasive infections. Pneumonia and bloodstream infections are particularly important because of their well-documented association with increased mortality and adverse clinical outcomes [6,12,14]. A recent study from Türkiye further demonstrated the high mortality associated with carbapenem-resistant Gram-negative bloodstream infections in critically ill patients, emphasizing the importance of timely diagnosis and appropriate antimicrobial therapy [15].

More than 80% of cases were classified as healthcare-associated infections, emphasizing the ongoing importance of healthcare facilities as reservoirs for carbapenem-resistant *Klebsiella* spp. Prolonged hospitalization, intensive care unit exposure, and person-to-person transmission remain major drivers of dissemination. Therefore, sustained implementation of infection prevention and control measures remains essential for limiting transmission within healthcare settings [10,16].

The extensive use of invasive medical devices observed in this study is another noteworthy finding. Urinary catheters, central venous catheters, and mechanical ventilation were common among affected patients. These devices are recognized risk factors for carbapenem-resistant *Klebsiella* infections because they facilitate biofilm formation and device-associated colonization, thereby increasing the likelihood of subsequent infection [11,13,16].

The observed colistin resistance rate was 23.5% (95% CI, 9.56–47.26). Although the confidence interval is wide because of the limited number of isolates tested, this finding remains clinically concerning. Although colistin has long served as a last-line therapeutic option for infections caused by carbapenem-resistant Gram-negative bacteria, increasing resistance rates have been reported worldwide. Similar findings have been documented in studies from Türkiye, suggesting a gradual decline in the effectiveness of colistin against multidrug-resistant *Klebsiella* isolates [9,17].

Another notable finding was the high rate of ceftazidime–avibactam resistance, with resistance detected in 12 of the 20 isolates tested (60.0%; 95% CI, 38.66–78.12). This finding should be interpreted in the context of previous clinical studies from OXA-48-endemic regions in Türkiye, where ceftazidime–avibactam treatment has become an important therapeutic option despite the emerging challenge of resistance [18]. During the study period, routine ceftazidime–avibactam susceptibility testing was not uniformly implemented across participating centers because local laboratory practices and national reimbursement policies limited its routine use in some hospitals. Nevertheless, recent data from Türkiye indicate that ceftazidime–avibactam resistance is becoming increasingly common among carbapenem-resistant *K. pneumoniae*. Eren-Kutsoylu et al. reported a resistance rate of 54.3% and demonstrated frequent detection of *bla*NDM, either alone or in combination with *bla*OXA-48, among ceftazidime–avibactam-resistant isolates [19]. More recently, Yanık and Karaşahin showed that ceftazidime–avibactam resistance in carbapenem-resistant *K. pneumoniae* bloodstream infections was associated with adverse clinical outcomes, underscoring the importance of preserving the activity of this agent through appropriate antimicrobial stewardship [20]. Furthermore, a recent multinational genomic surveillance study by Budia-Silva et al. demonstrated marked regional differences in the molecular epidemiology of carbapenem-resistant *K. pneumoniae* across Southern Europe and identified OXA-48-like-producing ST14 as the predominant lineage in Türkiye, whereas KPC-producing ST258/512 lineages predominated in Greece, Italy, and Spain [21]. These observations indicate that regional carbapenemase epidemiology may substantially influence ceftazidime–avibactam susceptibility patterns and should be considered when interpreting local resistance data. Because comprehensive molecular characterization was not routinely available in the present study, the specific mechanisms responsible for ceftazidime–avibactam resistance could not be determined. Previous studies have demonstrated that ceftazidime–avibactam-resistant isolates may exhibit distinct microbiological and clinical characteristics, further highlighting the importance of routine molecular surveillance and susceptibility testing [19]. Therefore, future multicenter surveillance studies incorporating standardized molecular testing are warranted to better define the molecular epidemiology and resistance mechanisms of ceftazidime–avibactam-resistant *Klebsiella* spp. in Türkiye.

Only one isolate underwent molecular characterization, and KPC production was identified in that isolate. The limited availability of molecular characterization data reflects routine laboratory practices in several participating centers, where comprehensive carbapenemase genotyping is not routinely performed. Previous studies from Türkiye have demonstrated that OXA-48 remains the predominant carbapenemase among carbapenem-resistant *Klebsiella pneumoniae*, although other carbapenemases, including NDM and KPC, have also been reported [22,23]. More recent data also suggest increasing molecular diversity among carbapenem-resistant Enterobacterales in Türkiye, including isolates co-producing multiple carbapenemases [24]. Therefore, the present study primarily describes the clinical and epidemiological burden of carbapenem-resistant *Klebsiella* spp. rather than the molecular distribution of resistance determinants. This limitation highlights the need for broader implementation of molecular diagnostic techniques within national antimicrobial resistance surveillance programs [20,21,22,23,24,25].

Another important observation was that only two participating centers reported routine screening for carbapenem-resistant Enterobacterales. Active surveillance, patient cohorting, contact precautions, and antimicrobial stewardship programs remain key strategies for preventing the spread of resistant microorganisms. Although isolation precautions were implemented in the majority of patients, variability among participating centers suggests that further standardization of national infection control policies may be beneficial.

From a clinical perspective, our findings highlight the ongoing burden of carbapenem-resistant *Klebsiella* spp. in tertiary-care hospitals in Türkiye. The predominance of critically ill patients, the frequent use of invasive medical devices, and the severe clinical condition of the study population emphasize the importance of early microbiological diagnosis, timely implementation of appropriate antimicrobial therapy, and strict infection prevention and control measures. Furthermore, the high rate of ceftazidime–avibactam resistance among the tested isolates underscores the need for routine antimicrobial susceptibility testing, careful antimicrobial stewardship, and continuous national surveillance to preserve the effectiveness of newly introduced therapeutic agents.

A major strength of this study is its multicenter design, encompassing tertiary-care hospitals from different geographical regions of Türkiye. However, several limitations should be acknowledged. First, the cross-sectional point-prevalence design provides only a single-day epidemiological snapshot and therefore does not permit assessment of temporal changes or longitudinal trends in antimicrobial resistance. Nevertheless, point-prevalence surveys represent a standardized and pragmatic approach for multicenter surveillance and provide valuable baseline epidemiological data. Future longitudinal multicenter surveillance studies are required to evaluate changes in antimicrobial resistance patterns over time.

Second, the relatively small number of included patients limits the statistical power of the study and the generalizability of the findings. Nevertheless, this reflects the inherent nature of a multicenter point-prevalence design, in which all eligible cases identified on the predefined survey day are included rather than a predefined sample size. Therefore, the findings should be interpreted as a contemporary epidemiological snapshot rather than definitive national estimates. Larger prospective multicenter surveillance studies are warranted to validate these observations.

In addition, because this study included only patients with carbapenem-resistant *Klebsiella* spp. identified during the predefined point-prevalence survey and did not include a comparator group of patients without carbapenem-resistant *Klebsiella* spp., analyses aimed at identifying independent risk factors or predictors were not methodologically appropriate. Accordingly, the findings should be interpreted as descriptive epidemiological observations rather than as evidence of causal associations. Future case–control or prospective cohort studies including appropriate comparator populations are needed to identify independent determinants and risk factors associated with carbapenem-resistant *Klebsiella* spp. acquisition and infection.

Third, molecular characterization was available for only one isolate, in which KPC carbapenemase was detected. This represents one of the major limitations of the present study. Since this was a multicenter study reflecting routine clinical practice, comprehensive carbapenemase genotyping was not routinely available at all participating centers during the study period; therefore, systematic molecular characterization could not be performed. Consequently, the present study was designed to describe the clinical and epidemiological characteristics of carbapenem-resistant *Klebsiella* spp. rather than to investigate their molecular epidemiology. Future multicenter surveillance studies incorporating standardized molecular testing across participating centers are warranted to better define the distribution of carbapenemase genes and resistance mechanisms in Türkiye.

As summarized in Table 7, hospital-level characteristics and infection prevention practices varied across participating centers, particularly regarding routine CRE screening and antimicrobial stewardship implementation. However, because only two to six cases were identified in each center, meaningful statistical comparisons were not feasible. Future multicenter surveillance studies including larger numbers of isolates from each institution will be better suited to evaluate regional differences in antimicrobial resistance patterns, infection prevention practices, and antimicrobial stewardship implementation.

Finally, the culture-based prevalence estimates reported in this study should be interpreted within the context of the study design. Microbiological cultures were obtained only when clinically indicated rather than systematically from all hospitalized patients. Therefore, using all hospitalized patients as the denominator would likely underestimate the occurrence of carbapenem-resistant *Klebsiella* spp., whereas restricting the analysis to cultured patients may introduce selection bias. Accordingly, these estimates do not represent patient-level prevalence but rather culture-based prevalence, providing descriptive epidemiological indicators of the burden of carbapenem-resistant *Klebsiella* spp. within the participating hospitals.

Another limitation relates to colistin susceptibility testing. Although broth microdilution is the EUCAST-recommended reference method, one participating center used the E-test because broth microdilution was not available at the time of the point-prevalence survey. As the isolates were not preserved after completion of the survey, confirmatory broth microdilution testing could not be performed retrospectively. Therefore, the reported colistin susceptibility results should be interpreted with caution.

Future studies incorporating larger sample sizes, systematic molecular characterization, and longitudinal surveillance data are needed to provide a more comprehensive understanding of the epidemiology and resistance mechanisms of carbapenem-resistant *Klebsiella* spp. in Türkiye.

The findings of this multicenter point-prevalence study provide contemporary epidemiological data that may strengthen national antimicrobial resistance surveillance and inform evidence-based infection prevention and antimicrobial stewardship strategies in Türkiye.

## 4. Materials and Methods

### 4.1. Study Design and Setting

This multicenter cross-sectional point-prevalence study was designed to evaluate the occurrence of carbapenem-resistant *Klebsiella* spp. and their associated clinical characteristics. The study was conducted in tertiary-care and teaching hospitals located in different geographical regions of Türkiye, including the Aegean, Central Anatolia, Mediterranean, and Marmara regions.

Participating hospitals were selected on the basis of their willingness to participate, the availability of infectious diseases specialists responsible for local data collection, and representation of different geographical regions of Türkiye. This approach was intended to provide a multicenter overview of carbapenem-resistant *Klebsiella* spp. across tertiary-care hospitals rather than to obtain a nationally representative sample.

### 4.2. Study Population

A point-prevalence assessment was performed simultaneously in all participating centers on 19 February 2026. All adult patients (≥18 years of age) who were hospitalized in inpatient wards or intensive care units on the study day were screened. Among these patients, those with at least one culture yielding carbapenem-resistant *Klebsiella* spp. from specimens collected on the study day were included in the study.

### 4.3. Data Collection

Hospital-level, ward-level, and patient-level data were collected using a standardized case report form.

Hospital-level variables included hospital type, institutional status, teaching affiliation, total bed capacity, number of intensive care unit beds, annual patient admissions, availability of a microbiology laboratory, antimicrobial stewardship programs, infection control committee activities, CRE screening policies, and isolation practices.

Ward-level variables included ward type, bed capacity, number of hospitalized patients at the time of the survey, isolation measures, and antimicrobial use protocols.

Patient-level variables included demographic characteristics, comorbidities, recent hospitalization history, prior antibiotic exposure, and current infection status. Healthcare-associated infections were classified according to the case definitions described in the European Centre for Disease Prevention and Control (ECDC) Point Prevalence Survey (PPS) protocol (Version 6.1) [7].

### 4.4. Microbiological Evaluation

Microbiological variables included specimen type, carbapenemase type, and classification of isolates as either colonization or infection. Colonization cases were not considered infections. Information regarding antimicrobial treatment, treatment indication, treatment duration, presence of invasive devices, and isolation measures was also recorded.

Species identification was performed using matrix-assisted laser desorption/ionization time-of-flight mass spectrometry (MALDI-TOF MS) (MALDI Biotyper, Bruker Daltonics, Bremen, Germany), VITEK^®^ 2 (bioMérieux, Marcy-l’Étoile, France), or BD Phoenix™ M50 (Becton, Dickinson and Company, Sparks, MD, USA), according to the routine laboratory practices of the participating centers. Antimicrobial susceptibility testing was performed using VITEK^®^ 2 (bioMérieux, Marcy-l’Étoile, France) or BD Phoenix™ M50 (Becton, Dickinson and Company, Sparks, MD, USA), in accordance with the routine laboratory procedures of each participating center. Colistin susceptibility testing was performed using broth microdilution in all participating centers except one, where gradient diffusion (E-test) was routinely used. Because ceftazidime–avibactam susceptibility testing was not routinely implemented at all participating centers during the study period, susceptibility results were available only for isolates tested according to local laboratory practices. Consequently, ceftazidime–avibactam susceptibility data were available for 20 isolates. Antimicrobial susceptibility results were interpreted according to the European Committee on Antimicrobial Susceptibility Testing (EUCAST) 2026 clinical breakpoints. Multidrug resistance (MDR) was defined according to the criteria proposed by Magiorakos et al. [26].

### 4.5. Statistical Analysis

All collected data were anonymized prior to analysis. Results were reported using culture-based prevalence estimates and descriptive statistics.

Categorical variables were expressed as frequencies and percentages. Continuous variables were assessed for normality using the Shapiro–Wilk test and visual inspection of histograms and Q–Q plots. Normally distributed variables were presented as mean ± standard deviation (SD), whereas non-normally distributed variables were expressed as median and interquartile range (IQR).

Culture-based prevalence estimates with 95% confidence intervals (95% CI) were calculated using the Wilson method in OpenEpi Version 3.0. All other statistical analyses were performed using IBM SPSS Statistics version 23.0 (IBM Corp., Armonk, NY, USA).

### 4.6. Use of Generative Artificial Intelligence

During manuscript preparation, a generative artificial intelligence (GenAI) tool (ChatGPT, OpenAI, GPT-5.5) was used exclusively to assist with English-language editing and improvement of sentence structure. No AI tool was used for study design, data collection, data analysis, interpretation of results, or scientific decision-making. All scientific content was reviewed, verified, and approved by the authors.

## 5. Conclusions

In conclusion, carbapenem-resistant *Klebsiella* spp. remain important healthcare-associated pathogens in Türkiye. The high prevalence of recent antibiotic exposure, extensive use of invasive medical devices, and substantial resistance to key antimicrobial agents emphasize the continued importance of infection prevention measures and antimicrobial stewardship programs. National surveillance efforts supported by comprehensive molecular epidemiological investigations are essential for accurately defining the burden of carbapenem-resistant *Klebsiella* spp. and guiding future control strategies.

## Figures and Tables

**Table 1:** Demographic and Clinical Characteristics of the Patients Included in the Study.

Variable	*n* (%) or Median (IQR)
Male sex	16 (61.5)
Age, mean ± SD (years)	68.5 ± 14.1
Presence of at least one comorbidity	22 (84.6)
Coronary artery disease	11 (42.3)
Diabetes mellitus	10 (38.5)
Chronic kidney disease	6 (23.1)
Cerebrovascular disease	4 (15.4)
Asthma/COPD	3 (11.5)
Solid organ malignancy	3 (11.5)
Chronic liver disease	2 (7.7)
Other comorbidities	13 (50.0)
Surgical ward	11 (42.3)
Medical ward	10 (38.5)
Intensive care unit	5 (19.5)
Length of hospital stay (days)	21.5 (2.8–48.3)
Hospitalization within previous 90 days	11 (42.3)
ICU admission within previous 90 days	8 (30.8)
Antibiotic use within previous 30 days	23 (88.5)

**Table 2:** Characteristics of Infections.

Variable	*n* (%)
Urinary tract infection	14 (53.8)
Pneumonia	5 (19.5)
Bloodstream infection	5 (19.2)
Surgical site infection	2 (7.7)
Other infections	2 (7.7)
Healthcare-associated infection	21 (80.8)

**Table 3:** Microbiological Specimens.

Specimen Type	*n* (%)
Urine	14 (53.8)
Respiratory specimen	7 (26.9)
Blood	5 (19.2)
Wound	1 (3.8)
Cerebrospinal fluid	1 (3.8)

**Table 4:** Invasive Device Use and Treatment Characteristics.

Variable	*n* (%) or Median (IQR)
Urinary catheter	19 (73.1)
Duration of urinary catheter use (days)	23.0 (8.0–30.0)
Central venous catheter	12 (46.2)
Duration of central venous catheter use (days)	21.0 (9.3–55.0)
Mechanical ventilation	9 (34.6)
Duration of mechanical ventilation (days)	25.0 (8.0–73.5)
Targeted therapy *	14 (60.9)
Empirical therapy *	9 (39.1)

* Evaluated among the 23 patients who received antimicrobial therapy.

**Table 5:** Infection Control Measures and Antimicrobial Resistance Results.

Variable	*n* (%)
Isolation precautions implemented	24 (92.3)
Contact precautions	17 (65.4)
Single-patient room	10 (38.5)
Antibiotic use protocol available	22 (84.6)
Colistin-resistant	4 (23.5)
Ceftazidime–avibactam-resistant	12 (60.0)

Note: The resistance mechanism was identified in only one isolate, in which KPC carbapenemase was detected.

**Table 6:** Characteristics of participating centers and culture-based prevalence of carbapenem-resistant *Klebsiella* spp.

Variable	Median	Q1	Q3
Total adult bed capacity	731	678	986
Intensive care unit bed capacity	83	53	126
Total hospitalized patients in 2025	40,017	38,407	58,940
Total cultures obtained on study day	252	86	287
Culture-positive specimens on study day	40	16	73
Carbapenem-resistant *Klebsiella* isolates on study day	4	2	4
Prevalence among culture-positive specimens (%)	10.5	5.5	15.0
Prevalence among all cultures (%)	1.6	1.2	2.3

**Table 7:** Hospital characteristics, infection prevention practices, and distribution of carbapenem-resistant *Klebsiella* spp. across participating centers.

Participating Center	Region	Adult Beds	ICU Beds	Annual Adult Admissions	Cultures Obtained on Study Day	Culture-Positive Specimens	CR *Klebsiella* spp. Isolates (*n*)	Prevalence Among Culture-Positive Specimens, *n*/N (%)	Routine CRE Screening	Routine Isolation Precautions	Antimicrobial Stewardship Program
Manisa Celal Bayar University	Aegean	731	83	38,407	86	14	2	2/14 (14.3)	No	Yes	Yes
İzmir University of Economics Medical Point Hospital	Aegean	281	53	10,789	52	16	4	4/16 (25.0)	Yes	Yes	Yes
Eskişehir Osmangazi University	Central Anatolia	809	61	40,017	287	76	4	4/76 (5.3)	No	Yes	Yes
Kocaeli University	Marmara	678	40	46,970	165	19	2	2/19 (10.5)	No	Yes	No
Çukurova University	Mediterranean	697	126	39,227	286	40	6	6/40 (15.0)	No	Yes	Yes
Dokuz Eylül University	Aegean	986	103	58,940	412	73	4	4/73 (5.5)	No	Yes	Yes
Ege University	Aegean	1806	130	67,966	252	53	4	4/53 (7.5)	Yes	Yes	Yes

Abbreviations: ICU, intensive care unit; CRE, carbapenem-resistant Enterobacterales.

## Data Availability

The data presented in this study are available from the corresponding author upon reasonable request. The data are not publicly available due to privacy and ethical restrictions.
